# Complex interplay between the microfluidic and optical properties of *Hoplia* sp*. beetles*

**DOI:** 10.1186/s12983-024-00552-0

**Published:** 2024-11-14

**Authors:** Danica Pavlović, Branislav Salatić, Srećko Ćurčić, Petar Milovanović, Dejan V. Pantelić

**Affiliations:** 1 Institute of Physics, University of Belgrade, Pregrevica 118, 11080 Belgrade, Serbia; 2 Institute of Zoology, University of Belgrade - Faculty of Biology, Studentski Trg 16, 11000 Belgrade, Serbia; 3 Laboratory for Anthropology and Skeletal Biology, Institute of Anatomy, University of Belgrade – Faculty of Medicine, Dr Subotića 4/2, 11000 Belgrade, Serbia; 4 Senzor INFIZ, Pregrevica 118, 11080 Zemun, Belgrade, Serbia

**Keywords:** Scarab beetle, Insect colors, Photonic structures, Functional morphology, Camouflage

## Abstract

**Background:**

All living organisms exist in a world affected by many external influences, especially water and light. Photonic nanostructures present in certain insects, have evolved over time in response to diverse environmental conditions, facilitating communication within and between species, camouflage, thermoregulation, hydration, and more. Up to now, only a few insect species have been discovered whose elytron changes its color due to permeation of water (or its vapor) through cuticle.

**Results:**

Here we report on a scarabaeid beetle *Hoplia argentea* remarkable in its ability to shift from green to brownish-red when exposed to water, demonstrating reversible changes. Here we show that elytron and scales form a complex and efficient micro/nano-optofluidic system. Water is channeled into the elytral lacunae, then transported internally to the petals of the scales, where it is wicked inside each scale, pushing the entrapped air out. Wicking is a very fast process, occurring during a few seconds. The advantage of this principle is in extremely high pressure (approximately 15 bar) produced by capillary forces, which expediates permeation of air. We present optical models that explain the physical mechanisms behind the coloration, detailing how superhydrophilic properties influence optical behavior.

**Conclusion:**

Species within the genus *Hoplia* exhibit diverse coloration strategies, likely linked to their specific ecological niches. These organisms have evolved intricate optical and microfluidic systems that facilitate rapid alterations in body coloration, potentially serving purposes such as environmental camouflage and thermoregulation. Studying microfluidic and optical properties of the elytra will not only enhance our understanding of the biological purposes behind color change but also inspires design of artificial biomimetic devices. Dynamic fluid flow patterns, described in this paper, are fairly constant and unique and can be used in security applications as a, so called, physically unclonable functions (PUF). More broadly, this kind of microfluidic system can be used for controlled drug release, sensing, hydraulic and pneumatic pumping.

**Supplementary Information:**

The online version contains supplementary material available at 10.1186/s12983-024-00552-0.

## Background

A number of mechanisms was evolutionary developed to counteract negative impacts of simultaneous action of external forces and enable survival in a hostile environment. Water and light are maybe the most important environmental factors and organisms must provide mechanisms to use them for their own benefit. This is especially true for insects due to their generally small size and variable environments. Some of them, like tortoise beetle (Cassidinae), provide a curious interplay between optics and microfluidics enabling color change upon interaction with water [1].

Interaction with water is the common problem of plants and animals, which is solved in different ways in different organisms (examples of an Australian Thorney devil lizard which wicks water through its body [2] or an African, long-legged, Namib desert beetle (genus *Stenocara*) which collects water on a hydrophilic part of its elytra [3].

Surface effects (capillarity and surface tension) are extremely important and affected by the integument structure and material properties. The final result can be often seen as hydrophobic or hydrophilic properties of the organism surface. Depending on the contact angle (between the water and the substrate), two extremes have been observed: superhydrophobicity (for contact angles larger than 100 deg–a classic example being Lotus leaf or rose petal [4] and superhydrophilicity (for small contact angles–e.g. [5]). Both effects are widespread not only in plants, but also in animals.

The adaptive advantages of these mechanisms are related to camouflage, signal communication, species recognition, thermoregulation or hydration [6–9], and they are caused by the organism's need to constantly adapt to the changing environment. Particularly interesting is the influence of water and humidity on optical properties (color) of an organism. Color change can be reversible or irreversible [10]. Reversible color change can occur due to changes in pigments, microstructure or their combination [11]. Many members of the Scarabaeidae family have the ability to structurally change color. Beetle *Pyronota festiva* (Fabricius, 1775) is usually green in color, but it can be purple, blue, red or brown, depending on the degree of hydration of the cuticle layers, by which it achieves better camouflage [12]. Some butterflies have the ability to change color quickly, which makes it difficult for predators to track them [13]. The level of hydration can change the thickness of the packed cuticle layers and thus change the effective refractive index of these porous layers—hygrochromism. Such a color change is known in the species *Hoplia coerulea* (Drury, 1773) [8], *Charidotella sexpunctata* (Fabricius, 1781) [6] and *Dynastes hercules* (Linnaeus, 1758) [7], and has a role in camouflage, aposematism or thermoregulation.

The vivid structural blue coloration and hygrochromism of *H. coerulea* beetle is well known and investigated [8, 14–16]. *Hoplia argentea* (Poda, 1761) (Insecta: Coleoptera: Scarabaeidae: Melolonthinae) is yet another species that attracted attention of scientist. The first characterization of the physical mechanisms lying behind the structural coloration is given by [17].

Here we report on wicking properties of *H. argentea*, a species of the scarabaeid family which, upon contact with water, switches its greenish color to brown-reddish. We microscopically analyzed its anatomy, observed the color change spectroscopically and provided mathematical models to explain how the water comes in its wing scales and dramatically changes its color. *H. coerulea* was analyzed too, to show that slight anatomical differences between *H. argentea* produce remarkable optical and microfluidic differences. Biological role and adaptive advantage of the changes in optical patter were discussed and possible reasons why two similar species, living in Europe and sharing similar habitat, develop two very different optical signaling mechanisms.

## Results

### Comparative optical and morphological analysis of *H. argentea* and *H. coerulea*

**Fig. 1:**
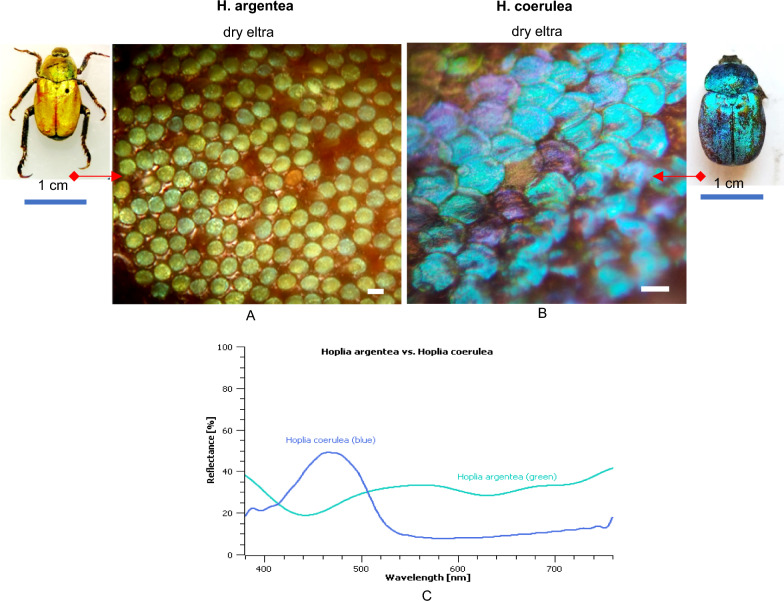
Closer look of elytra with scales, in dry condition of both *Hoplia* species. **A**. *H. argentea*. **B**. *H. coerulea*; scale bars are 50 um. **C**. Reflectances of dry *H. argentea* and *H. coerulea*

*H. argentea* adults have body lengths of 9 -12 mm [18] covered with green scales (Fig. 1A inset). The dorsal side of the upper wings (elytra) are dull green with no iridescence. The closer look reveal that round shaped scales are not tightly packed and we can see the brown-reddish surface of the elytron below (Fig. 1A). Also, there are a lot of scratched parts of the elytra, due to rubbing against vegetation.

On the contrary, *H. coerulea* is known for its striking iridescent blue coloration, that comes from overlapped, round shaped, blue scales that are tightly packed, completely covering the surface of the elytra (Fig. 1B). For this reason, under different lighting angles, the insect can appear from whitish to intense blue.

Reflection spectra were measured for both species and correlates with those found in literature [8, 15, 17]: *H. argentea* is characterized by a pronounced reflection peak at approximately 550 nm which is responsible for greenish coloration. However, there is a broad reflectance from 450 to 600 nm which de-saturates the observed coloration. Perceptually, such spectrum correlates well with the green-yellowish appearance of the insect. Nevertheless, the spectrum of the elytra itself, breaking through between the scales, affects the entire spectrum (Fig. 1C).In addition, there are slight differences in reflectance spectra of different individuals (as well as in different spots of the samespecimen) due to scale loss. Dark brown elytron under the scales has a reflectance ∼20% at visible wavelengths, showing exponential increase towards the IR part of the spectrum, indicative of the presence of a broadband-absorbing pigment, probably melanin [19]. Blue *Hoplia* as expected has reflectance shifted towards blue part of the spectrum and the higher intensity of reflectance.

Dull green coloration of *H. argentea* arises mostly from reflection from the individual scales. Even when we remove them from elytra, individual scales stay greenish. By immersing the single scale in an index matching liquid (manufactured by Cargile, series A, with the certified refractive index of 1.5700 ± 0.0002) Fresnel reflection was suppressed. If observed in transmission, an individual scale has slight residual absorption. In reflection, after some time, the scale removed from elytra, becomes completely invisible, upon index matching fluid uptake proving that the nature of the color is structural [8].

**Fig. 2:**
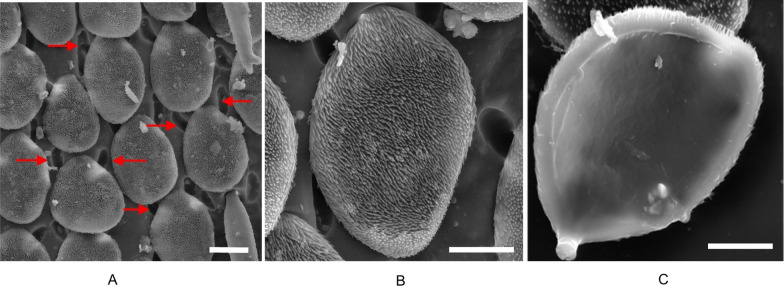
Micron-sized structure of *H. argentea* observed under an SEM microscope. **A**. scales on the elytra surrounded with openings (designated with red arrows) **B**. Patterned dorsal side of scale. C. Almost flat ventral side of a wing scale. Scale bars are 20 µm

**Fig. 3:**
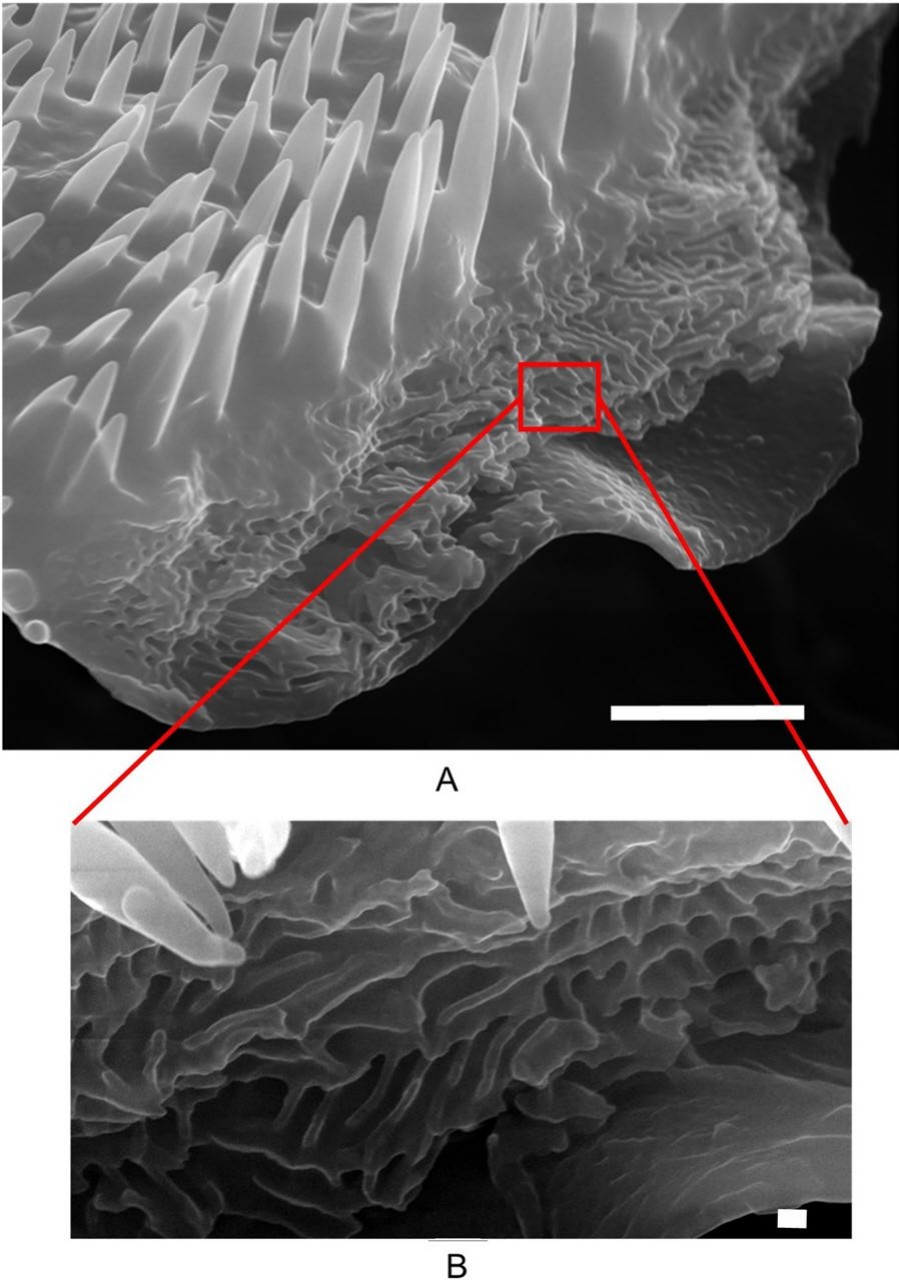
Ultrastructure of *H. argentea* (broken) scale as observed under an SEM microscope. **A**. Tiny fiber-like thorns cover the dorsal side while; scale bar is 2 µm. **B**. Internal structure of the scale is characterized with an array of tiny channels; scale bar is 100 nm

Analyzing the morphological and ultrastructural features, we can see that scales are attached with their peduncle on the distal part to the elytra (Fig. 2). SEM micrographs reveal their position, size and shape. Scales are oval, concave, about 60 µm long, 40 µm wide, and 3–4 µm thick. The upper (dorsal) surface of the scales is densely covered with vertically arranged filamentous top layer, each (hair-like) filament being 1–4 µm long and about 500 nm thick at its bottom (Fig. 2B and 3A). The lower (ventral) surface of the scales is completely smooth and almost flat (Fig. 2C). After trying to mechanically remove the scales, some of them were broken, revealing the internal structure. From these micrographs (Fig. 3B), a larger number of irregularly stacked layers, roughly parallel to the surface of the scale, was observed. The thickness of the individual layer was estimated at about 110 nm. Such architecture locally functions as Bragg's lattice and produces color by a coherent scattering mechanism. However, the general organization of each of these layers is quite disordered and looks more like an array of nanochannels meandering through the layers [8]. Each individual layer is characterized by a structured surface, which is built of a laterally distributed network of ridges, in the form of rods about 80 nm wide, separated with air voids of a thickness of about 90 nm, which further contributes to the scattering (Fig. 3B).

*H. coerulea* scales morphological features are described in details, in numerous papers [8, 15, 16]. The internal structure of the scales is practically the same as the one within the *H. argentea* scales, with stacked layers consisting of rods and air voids [8]. The obvious and most important difference is in the scale surface, which is completely flat and brushless in *H. coerulea*.

**Fig. 4:**
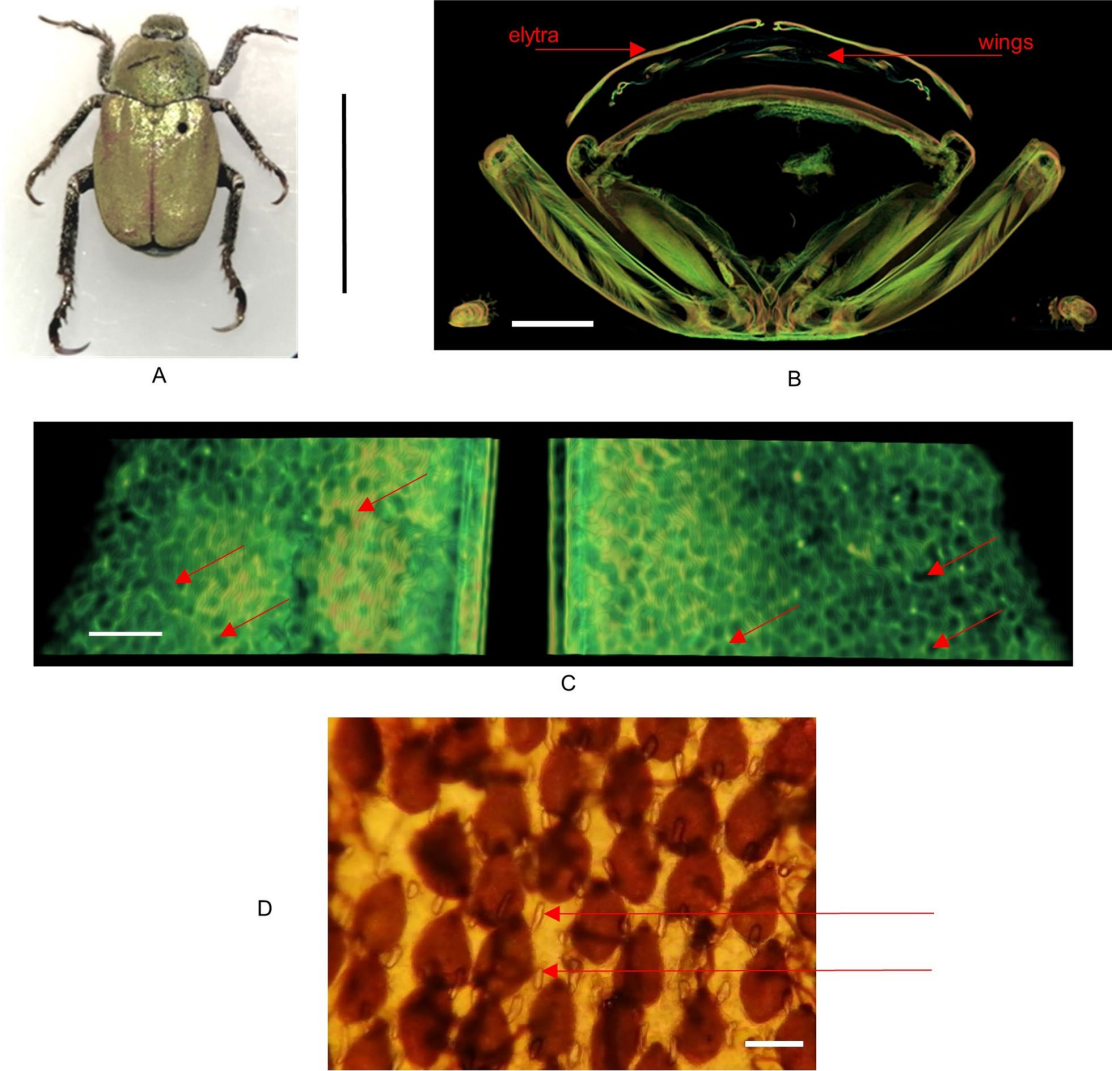
*Hoplia argentea.* **A**. Dry insect; scale bar 1cm. **B**. Micro-CT transverse cross section of the body, showing elytra and wings; scale bar 1 mm. **C**. Micro-CT image showing voids within the elytron (a few of them, seen as round dark areas, are designated with red arrows); scale bar 100 um. **D**. A transmission microscope image of *H. argentea* elytra, showing channels (red arrows indicate two, but more are visible, too) surrounding each wing scale; scale bar 50 um

**Fig. 5:**
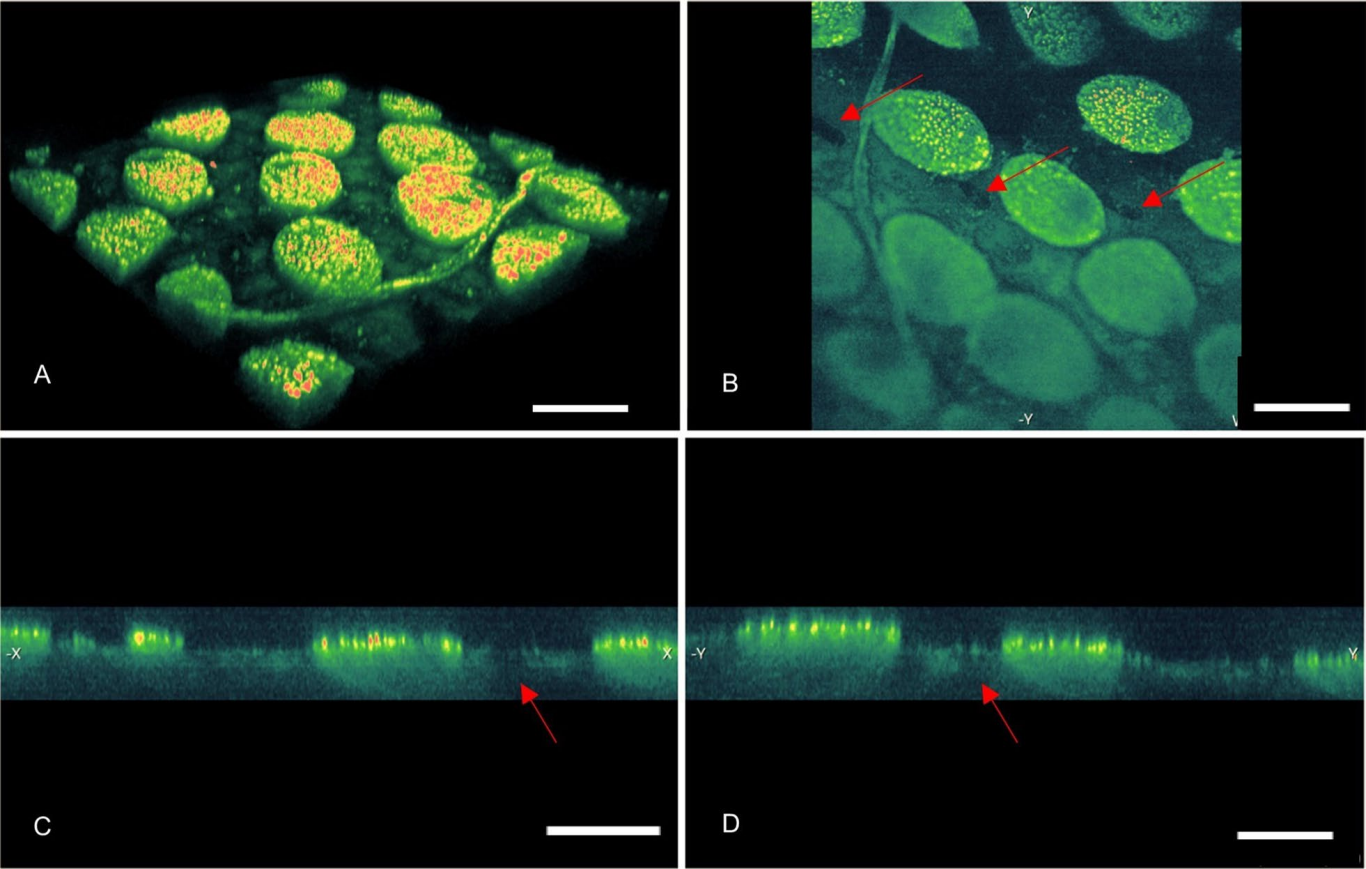
NLSM images of *H. argentea.* **A**. 3D-rendering of insect elytra. **B**, **C**, **D**. Transverse, sagittal and coronal planes, respectively, revealing that wing scales are in close contact with underlying elytron (red arrows indicate some of the openings); scale bars are 50 um

All research papers were focused on scales of both *Hoplia* species. None of them dealt with the analysis of the elytra itself. In *H. argentea,* elytra, under the scales, are smooth with numerous small openings surrounding scales (Fig. 2A). These opening are also visible when elytra are observed in transmission light (Fig. 4D). It looks like each scale is surrounded with two openings, one on each side of the peduncle. In order to find out what is on the other side of the openings, that is, inside the elytra, we performed micro-CT analysis. Micro-CT analysis showed that these openings lead to lacunae inside the elytra (Fig. 4B and 4C). To confirm that we did additional measurements using Nonlinear Laser Scanning Microscopy (NLSM) in order to get 3D-rendered, transverse, sagittal and coronal planes of elytra (Fig. 5A-D). These planes allow us to see cross section of the elytron, where we can see channels, which continue on elytral openings, leading to lacunes inside elytra (red arrows in Fig. 5B-D). Subsequent detailed microscopy analysis, with precise focus adjustment in the interior of the elytra, definitively confirmed the existence of the lacuna (see Fig. 9). We confirmed the existence of elytral openings and lacunae in *H. coerulea* as well.

### Microfluidics and liquid induced color change of *H. argentea*

As already mentioned, there are a number of papers dealing with *H. coerulea* color change upon wetting with different liquids [14]. The male individuals of this species change their coloration from blue to green upon scale water uptake. We noticed that *H. argentea* elytra changes color from green to reddish-brown upon wetting, therefore, we proceeded to a detailed analysis.

**Fig. 6:**
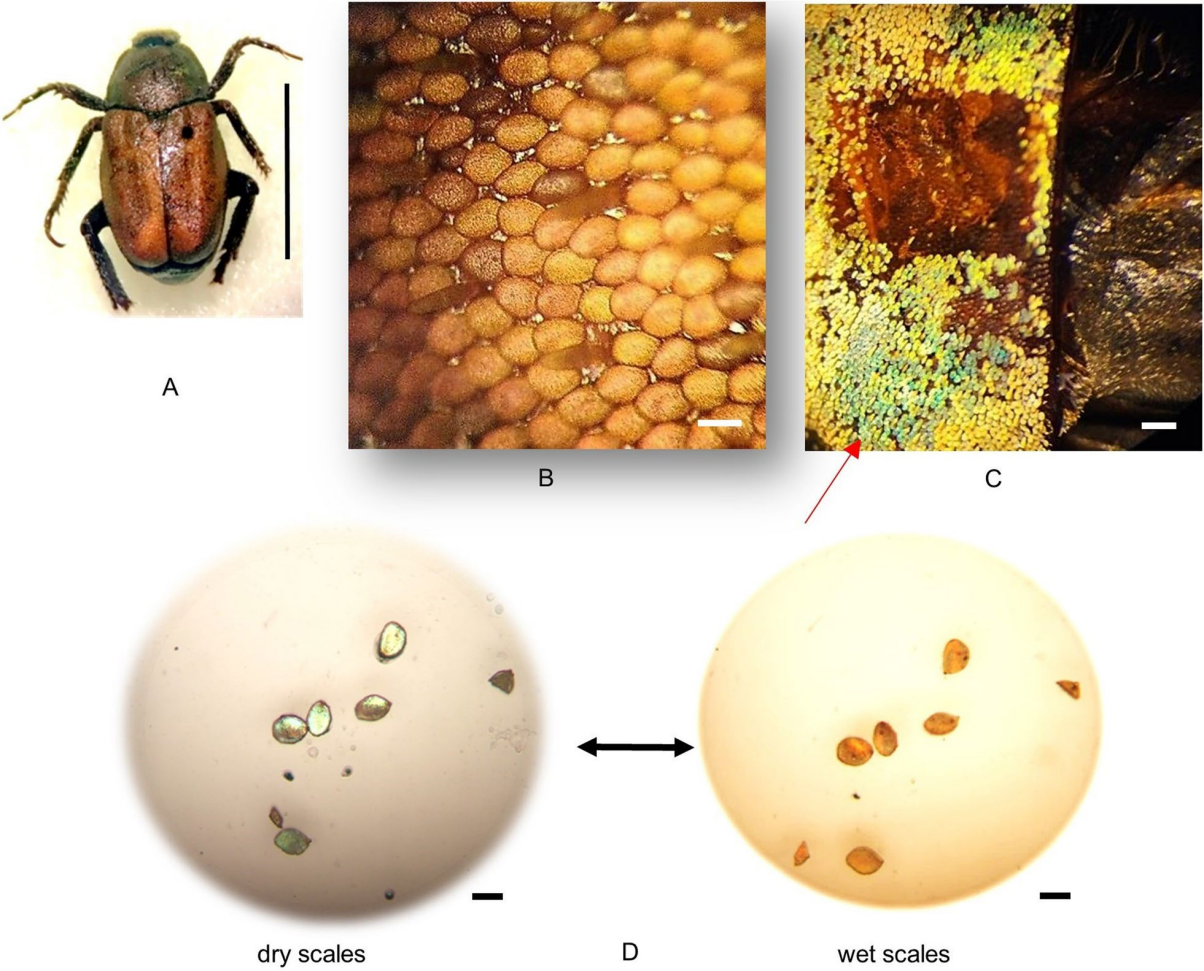
*H. argentea* (the same individual from Fig. 5A). **A**. Wet insect; scale bar 1 cm. **B**. Closer look at completely wet elytra and attached scales attaining brownish color; scale bar 50 um. **C**. Part of elytra immediately after applying a few droplets (each of 0.3 ul) of water with scales instantly turning greener; scale bar 1 mm. **D**. Individual scales of *H. argentea* (dry and wet). Arrows indicate the reversibility of the color change; scale bar 50 um

When the whole insect was submerged in water, the color change occurs relatively quickly (roughly 5–6 min) and insect turns brownish (Fig. 6A and B). This change is gradual, suggesting that it is the result of water absorption by scales. Also, the process is reversible, gradual and uneven along the elytra and it takes more than an hour at room temperature for the elytra to dry completely and regain its green color. But after about 30 min some of the scales start to regain green coloration.

Then we performed gradual wetting of the elytra. With successive wetting (droplet by droplet (0.3 µλ)), it seemed that, depending on the place on the elytra, the droplet of water was remained on the surface approx. 5 min and then it was imbibed by elytra. In contrast, the droplet on the elytra of *H. coerulea* remains on the surface indefinitely. The reason for this is the position of the scales, which in this species are densely packed, with no space between them, making the elytra superhydrophobic as in Morpho butterfly [20]. Thus, it takes more time for the water to penetrate the elytra.

Furthermore, addition of such a small amount of water on the elytra of *H. argentea*, did not make the scales turn brownish imediately, but more intensely green (Fig. 6C). Only after the elytra was soaked with a critical mass of water the scales turned brownish. That's why we decided to remove a certain number of scales and check their water absorbing properties, when they are not attached to the elytra. After immersing individual scales in the water, color change was observed in a few seconds (Fig. 6D). This change is reversible, and the evaporation of water from scale is as quick as absorption, when it runs out of water supply. Upon evaporation of water, scales regain their original, green color, which we confirmed through several cycles of wetting and drying of the elytra and individual scales.

**Fig. 7:**
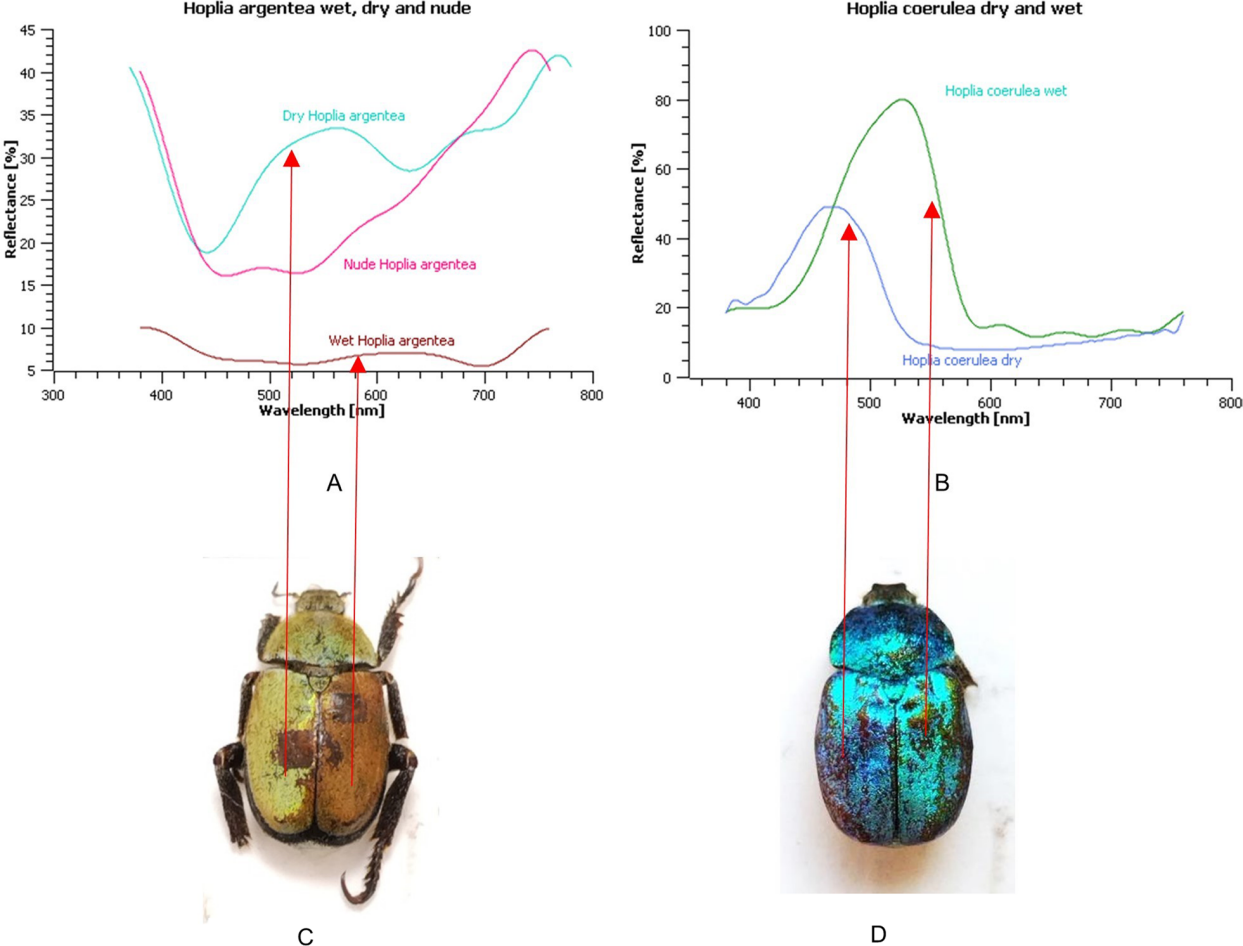
Reflectance spectra of two *Hoplia* species. **A**. Reflectances of *H.argentea* in dry and wet conditions, together with dry cuticle without scales (nude). **B**. Reflectances of *H. coerulea* in dry and wet conditions. **C**. Specimen of *H. argentea* with scales removed from a rectangular area to show the color of underlying elytra with one dry (left) and one wet (right) elytron. **D**. Specimen of *H. coerulea* with one dry and one wet elytron

The reflection spectra of dry and wet elytra were measured, as shown in Fig. 7A. Immersion in water shifts the peak towards red (650 nm), which correlates with the perceptual change in coloration. We have also measured the spectra for dry and wet elytra of *H. coerulea* (Fig. 6B). However, the difference in coloration of wet and dry elytron of blue *H. coerulea* is not so prominent as in *H. argentea*. We can observe, from different angles, that certain parts of elytra becoming green after wetting. Densely packed scales, which prevent the passage of water to the elytra, could be the reason. Also, the blue scales are iridescent, thus the coloration depends on the angle of observation / angle of incident light (the individuals lost their limbs during the manipulation).

As shown above, *H. argentea* scales possess multiscale patterns ranging from 80–100 nm up to several microns. It seems that they promote a quick optical response to water, which quickly turns a single scale from reflective to absorptive state.

**Fig. 8:**
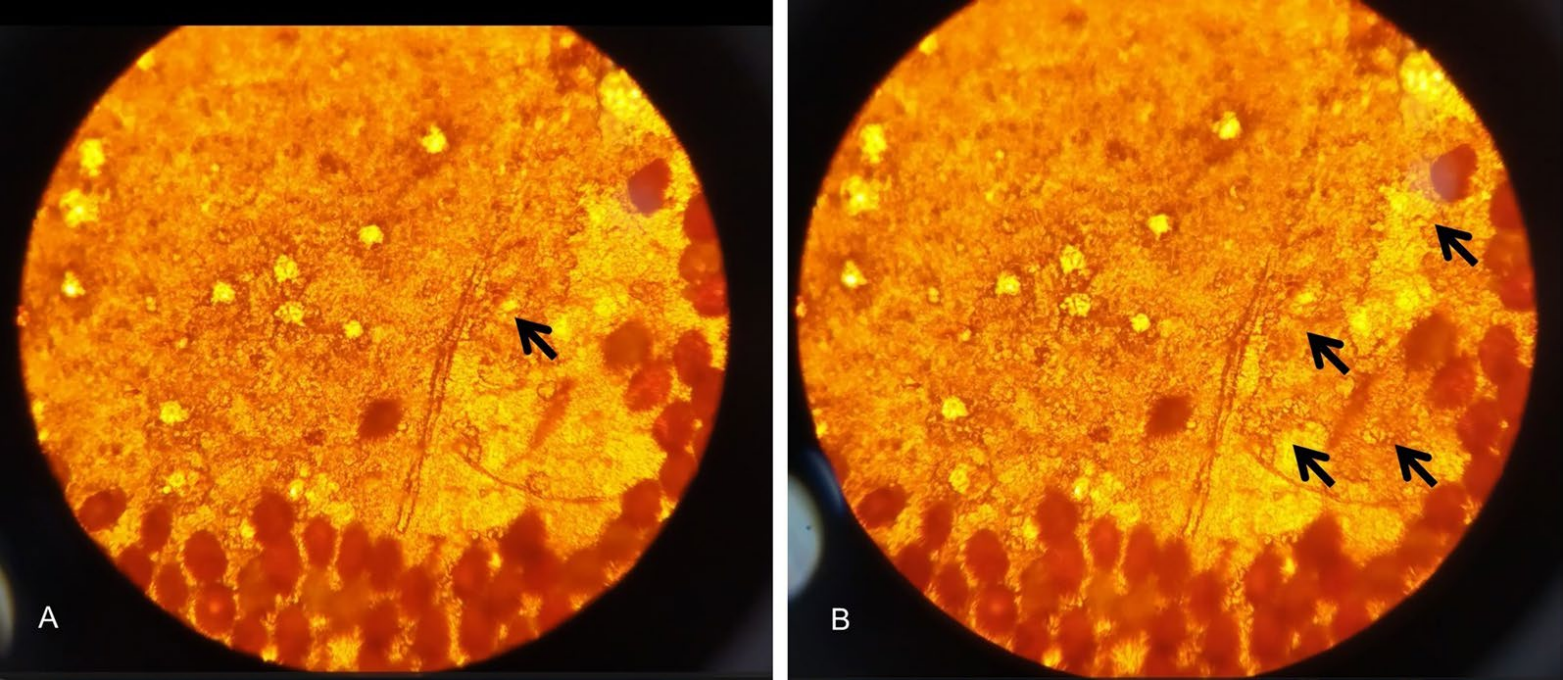
Transmission optical images of the water diffusion inside the lacuna system of *H. argentea* elytra. A and B. Pictures captured from the video (Supplementary Video S1) showing water spreading inside lacuna (arrows indicate successive path of water diffusion)

It seems that the whole micromechanical structure of scales and elytron is designed to use capillary forces to imbibe water very efficiently, making the insect surface behave, as a whole, in a superhydrophilic manner [21]. As we described, each scale lays flat on the cuticle and is surrounded by micron sized openings on both sides. It seems that openings lead deep into the lacunae of the underlying cuticle and are capable to introduce water into the elytron, followed by wicking into the embedded scale. We confirmed this recording water as it diffuses through a system of lacunae within the elytra (Fig. 8, Additional file 1: Supplementary Video S1).

Previously [14], it was proposed for *H. coerulea* that the water diffuses through the scale upper surface and fills the internal spongy structure. However, water is dense and it doesn’t explain the fast process of water-induced color change – which we observed to last a few seconds.

**Fig. 9:**
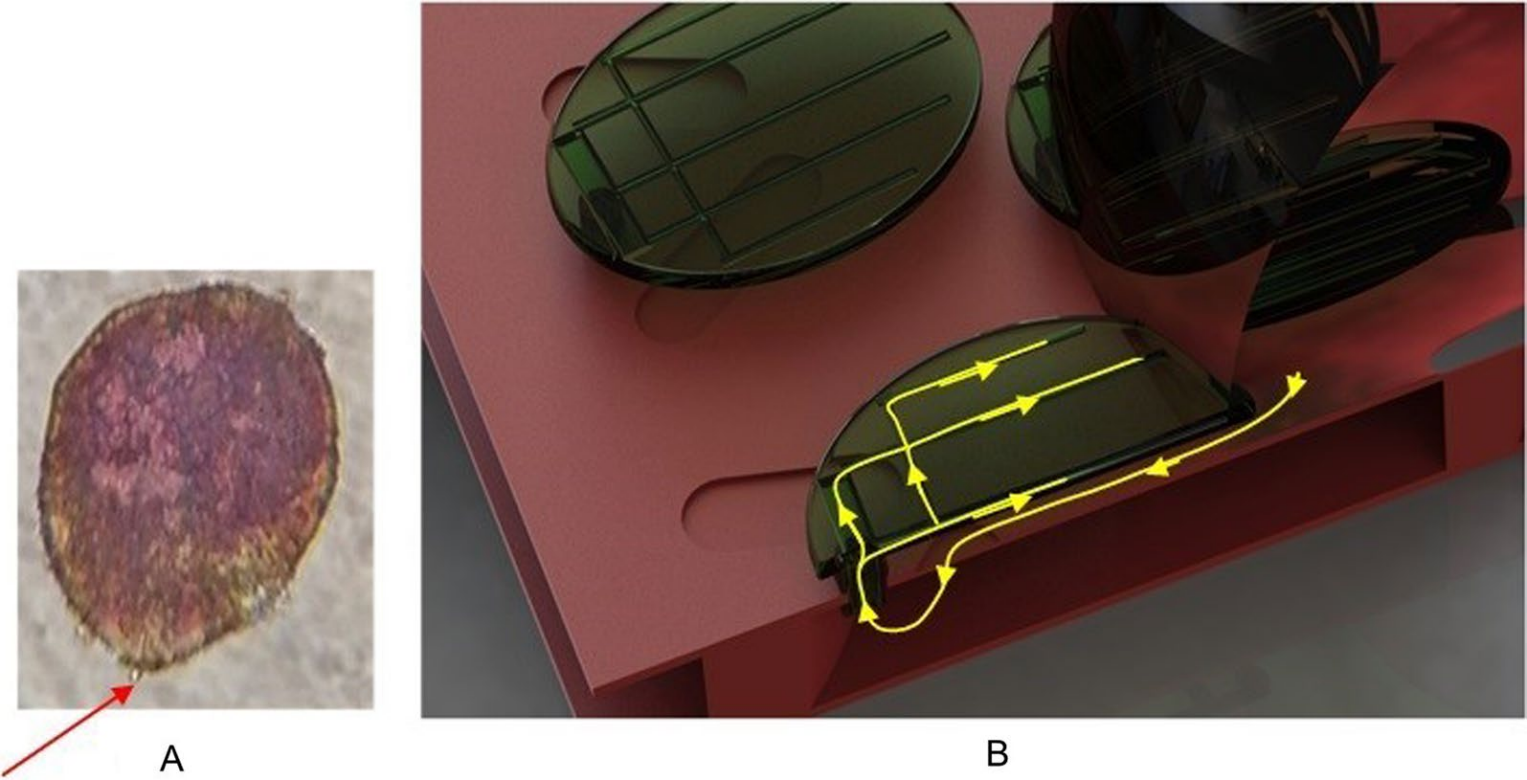
A single scale and a schematic model of its interior structure. **A** Original scale of *H. argentea (red arrows indicating the petal of the scale)*. **B** Microfluidic model of a scale on elytron. Yellow lines indicate the direction of water flow

It is much more probable that the process is enabled by the capillary action. From the microfluidic point of view, a wing scale is a system with an input channel (going through the petal—see red arrows in Fig. 9). This is the micron-sized channel (easily seen through the optical microscope, Fig. 9A) which branches into a number of nanoscale (approximately 110 nm diameter) channels seen in SEM images Fig. 3. This is a maize-like system difficult to analyze exactly. Essentially, a wing scale is a closed fluidic system with only one input port–petal of the scale. Therefore, we propose a simplified model depicted in Fig. 9B.

Our experiments have shown that the scale is filled with water quickly, in a matter of seconds. However, for 110 nm diameter channels, a model based on Young–Laplace equation for capillary pressure in a tube increase $\Delta p$ in a tube of radius *r* [22].

$$\Delta p = \frac{2\gamma \cos \left( \phi \right)}{r}$$

where γ is the surface tension and *ϕ* is the contact angle predicts the pressure of 150 kPa (15 bar) and millisecond filling time, for an estimated contact angle *ϕ* of 30–45 deg, and surface tension of water *γ* = 71.97 mN/m at 25 C. However, we have observed that each and every scale is completely filled by the water within much longer time frame (3–10 s)–as indicated by complete disappearance of structural color. This signifies that the air permeates through the walls of the scale partially dissolving in the water. This is a complex process (which includes air molecules absorption, diffusion and desorption, approximately described by Henry’s law [23]. We were able to establish the value the Permeability coefficient at 1.6 10^–11^ g/(m s Pa), in agreement with the air permeability of many polymers [24–27].

### Optical modeling

**Fig. 10:**
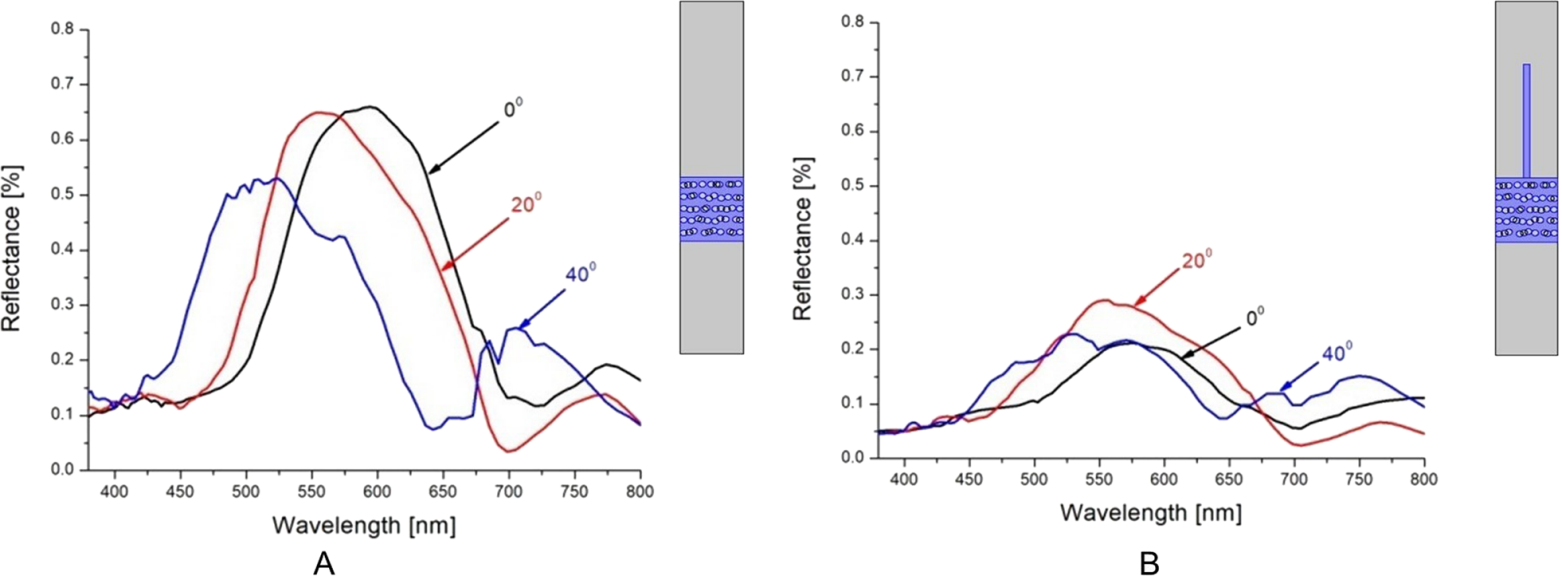
Calculated spectra for two models (without and with filaments). **A** Simple Bragg grating-like model, corresponding to *H. coerulea*–three different angles of incidence show pronounced iridescence. **B** Bragg grating with hair-like protrusions, corresponding to *H. argentea*–spectrum is largely insensitive to angle of incidence. The corresponding models are shown as insets on the right of each spectrum

In order to examine the physical mechanism behind the coloration of *Hoplia* species, we developed optical models that take into account the complex micro and nano structure of the *Hoplia sp.* scale. Mathematical calculation was done using Finite Element Method (FEM). As the main difference between the scales of two species, we wanted to evaluate the contribution of the filamentous top layer to the reflection spectrum. As a reference, we took a model without the filamentous top layer and internal structure retained (inset in Fig. 10A). The resulting spectrum (Fig. 10A) strongly shifts towards blue with the increasing illumination angle (iridescence), which correlates with the experimental measurements for blue *H. coerulea*, whose scales are brushless. Adding the filamentous top layer to the model surface (inset in Fig. 10B), produces spectra in the green spectral area, completely insensitive to illumination angle (Fig. 10B). Likewise, the reflectivity is much lower–less than 20%, compared to almost 60% for completely flat scale model, which is due to absorption by the filamentous structure. Details related to the optical model can be found in the Materials and Methods section.

**Fig. 11:**
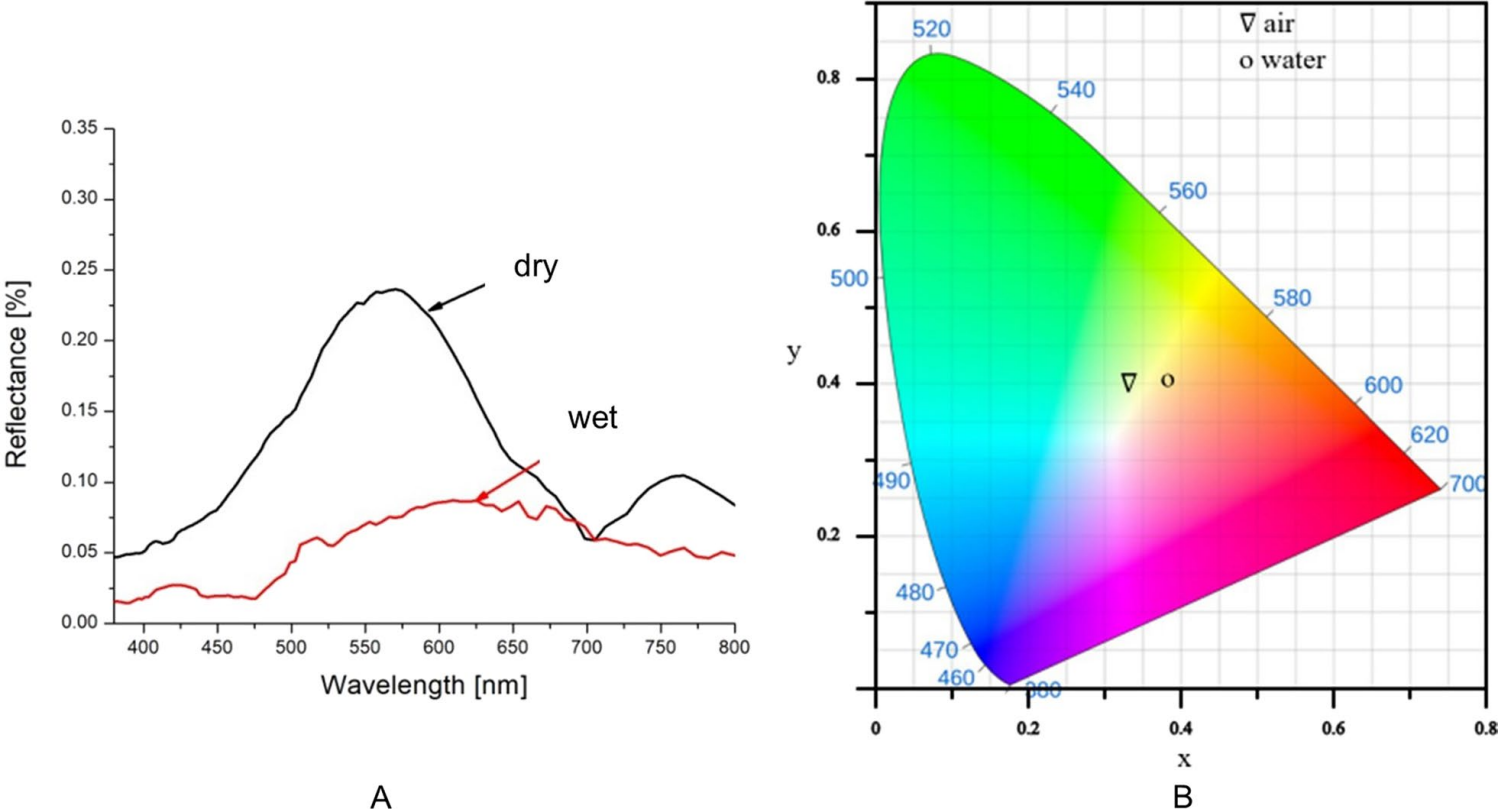
The averaged spectra and position of resulting colors in CIE diagram. **A**. Theoretical spectra of wet and dry elytra of *H. argentea*. **B**. CIE color system representation

The influence of water and humidity on the optical properties (color) of the *H. argentea* scales was other part of our modeling. Two cases were considered, when the structure from Fig. 10B is in air (simulating dry scale) and when it is immersed in water (simulating wet scale), implying that nanochannels inside the structure are filled with water. The simulation was done for 50 slightly different models (to take into account the natural variability of scales) and for three incidence angles 0°, 20° and 40°. The results were averaged and shown in Fig. 11A. From the figure it is clear that there is a shift in the reflection spectrum from the green to the red part of the spectrum, which is a consequence of the different indices of refraction of air (n = 1) and water (n = 1.33). For better visualization, these results are also shown in the CIE diagram (Fig. 11B).

**Fig. 12:**
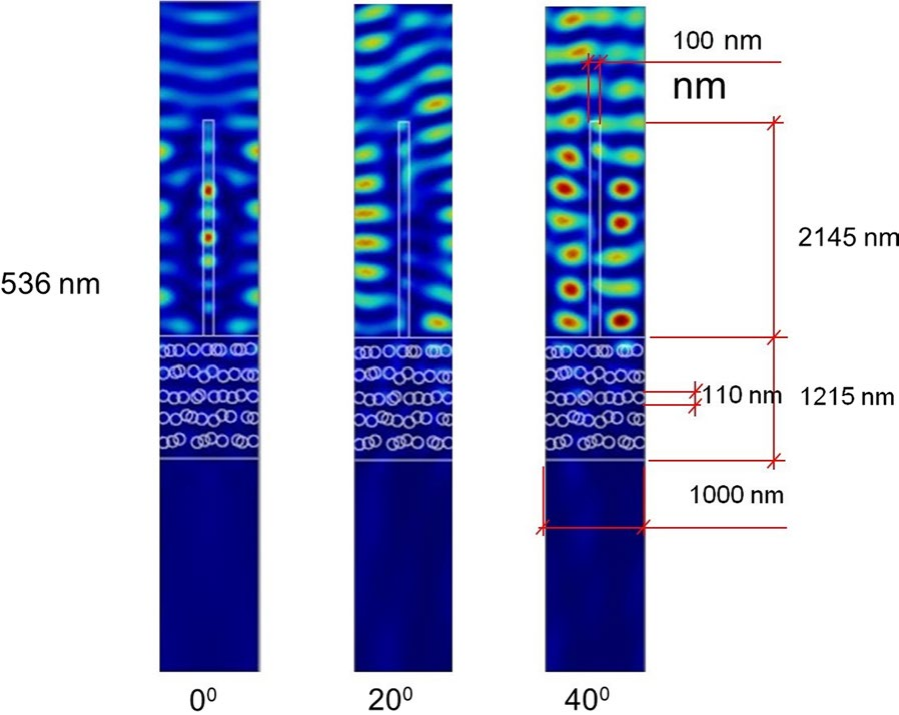
Field distribution within the scale of *H. argentea* shows waveguiding behavior of hairs

The distribution of electromagnetic radiation was calculated, too. It can be seen from Fig. 12 that radiation is guided through the filamentous top layer and absorbed inside it, due pigments. We assumed that absorption corresponding to melanin, one of the most abundant pigments in animals) [28]. The localization of electromagnetic radiation is observed in the entire spectrum of incident wavelengths, and one characteristic wavelength is shown in Fig. 12. The simulation also shows that the absorption depends on the morphology of the structure and the incident angle of irradiation. Localization of the electromagnetic field and absorption significantly affect the reflection spectrum of the structure, reducing the total reflection of the system, which can be seen from the theoretical spectrum in Fig. 10.

As a final remark, we have found that the same wing scale has quite different coloration depending on the side from which it is observed. Ventral side of the scale is dull green, while its underside has bright golden color. Our calculations and experiments show that the hairs are the main cause. If the single green scale is immersed in water in the first moment it becomes golden bright (Additional file 2: Supplementary Video S2). Afterwards, after imbibing water, all the structural coloration is lost and pigmentary brown-reddish color remains. From the theoretical point of view, it is understandable. If observed from the hairy side, radiation is guided and scattered before reaching the Bragg grating. If observing its underside directly, the surface is flat, without hairs, and the radiation is reflected from the Bragg grating before reaching the hairs. Finally, if wet, the effect of hairs is lost, due to the refractive index matching, and the Bragg grating reflects the radiation directly, before it is completely filled with water.

## Discussion and conclusions

Probably, the most popular and most studied *Hoplia* (Illiger, 1803) species is *Hoplia coerulea*, due to its iridescent bright blue coloration. This coloration arises from light interference within a chitinous macroporous photonic structure enclosed by a thin envelope, found inside the circularly shaped scales covering the beetle elytra [8]. The species has also been the subject of numerous studies due to its property of changing color from blue to green in contact with water and alcohols [14]. The green *H. argentea* has attracted attention for elytral scales similar to those of *H. coerulea* of which they differ by brush-like, diffusive layer on the upper side of the scales. The first time the difference in scales ultrastructure and optical properties between two species was pointed out by Gentil back in 1943 [29]. More detailed morphological and optical analysis of *H. argentea* scales was done recently [17]. The authors modelled optical properties of *Hoplia argentea* scale structure by assuming regular, layered, Bragg-like internal structure, and the upper brush- like layer of it. Based on focused ion beam (FIB) cross section data, authors used effective medium theory to approximate porous layers as a homogeneous one with refractive index 1.1. A transfer-matrix method was utilized to compute the reflection spectrum of a scale. However, the authors just mention the possibility of colour change due to wetting, on the basis of similarity with *H. coerulea*, but without further study.

However, it turned out that the wetting in this species is very interesting and it brings new view on hygrochromism of the species and its role. We discovered and examined a complex interplay between microfluidic and optical properties of the species elytral structures. One specimen of the species *H. coerulea* served us for comparative structural—optical analysis. Both scarab beetle species show hydrophilicity and change of elytral coloration with the accumulation of moisture, which shifts the reflection spectrum towards larger wavelengths. Wet *H. coerulea* elytra change coloration from blue to green [8], and *H. argentea* from green to brown-reddish. Previous results on blue *H. coerulea* wetting showed differences in the rate of color change of scales when soaked in different liquids [14, 15]. Likewise, [14]showed when spraying a 1 nl water droplet on a top side of a scale on cuticle, color changes occur. However, their SEM and TEM analyses did not show any micro or mesopores on the scale surfaces. Also, they did not manage to confirm by any method presence of nanopores. The explanation of authors is that presence of salts (NaCl, KCl and CaCl_2_) in the scales’ cuticle, revealed by X-ray photoelectron spectroscopy (XPS), as they say, “most likely foster the capillary flow of water into the micropores”, producing the hydrophilicity of the surface. Nevertheless, examining the morphology of the whole elytra and individual scales of similar *H. argentea* species, we discovered that they all together work as a superhydrophilic machinery, that absorbs water by capillary forces. Micron-sized openings on the elytra introduce water inside the elytra, where it diffuses over the internal lacunae. After the elytra is soaked with water, the embedded scales absorb water from it by the capillary forces of their peduncles and scale nanochanels. We have found experimental confirmation in structure’ morphology and drastically different speed rate of color change between the scales embedded into the cuticle and the scales removed from it. It takes several minutes for water to enter and soak elytra enough to come in contact with scales’ peduncles, after which it is imbibed into the scales’ nanochanels. Peeled individual scales that were put into direct contact with water change color very quickly (5–10 s).

We have measured experimentally and calculated theoretically the exact time of water intake by individual scale. Theoretical calculations include and explain the physics which lies behind this complex process, confirming experimental findings. Microfluidic model described here is simplified, disregarding randomized three-dimensional structure of nanochannels. Our goal was to understand the behavior in the most general terms and get some insight into interplay between capillary pressure and permeation through the walls of individual scales. More complex percolation model is needed to approximate a complex grid of nanochannels. As the matter of fact, we have observed characteristic patterns of percolation fingering during in- and out-flow of water (see Additional files 2, 3: Supplementary Videos S2 and S3). We did not perform all the microfluidic measurements to the blue *Hoplia* as well, but we are inclined to believe that the mechanism is the same, because the internal structure of the scales and the cuticle is almost identical. The only difference is in filamentous top layer of the scales.

We confirmed theoretically, that this top layer is responsible for the spectral differences between two *Hoplia* species. It shifts spectrum to the green part, making it insensitive to illumination angle, and suppressing the intensity of reflection. This is in complete agreement with the results from [17]. Additionally, our calculations and theoretical spectra confirmed that scales of *H. argentea* become brownish, when filled with water. This is achieved by refractive index matching, when the air layers inside the elytral scales are soaked with water. Thereafter scales become more transparent, incident light passes through the scales, reflects from elytral surface (which is brown) back through the scales. Furthermore, by applying finite element method we were able to see finer mechanism which uses filamentous top layer setae on the surface of each scale as tiny optical waveguides. They trap and guide light deeply into the structure, effectively increasing the optical path length and selectively absorbing certain part of the spectra better than others. Due to the adequate refractive index of chitin (n = 1.56), light is efficiently trapped inside filament fibers within the certain angular range. Slight tapering of each filament seta additionally improves the collection efficiency, similarly to apertureless scanning near-field microscope (SNOM) tips [30]. Consequently, radiation is absorbed more efficiently, in accordance with Yablonovich’s theory [31]. As a result, even a small amount of pigment (usually melanin) increases absorption significantly [19].

Species of the genus *Hoplia* (more than 250 species [18]) exhibit polychromatism, meaning that body coloration greatly varies (dark blacks and browns, through to bright blues, greens, yellows and orange), even within species and between the sexes [32]. In general, little is known about the biology of the species of this genus. Sexual dimorphism in *Hoplia* sp. is associated with display and mimicry [33].

*H. coerulea* is exceptional with iridescent blue coloration. It is not entirely clear what is the biological role of the bluish coloration. What is known is that the species lives in southwestern Europe (is considered a Franco-Iberian endemit [34] on green vegetation, near watercourses, where the soil is moist. Males swarm, while females are very rarely seen, usually near swarms of males and do not have such a striking coloration [32, 35]. Based on that, it is assumed that it is a signal coloring that serves in intraspecific recognition [8]. In *H. argentea* sexual dimorphism is not expressed, both sexes have a dull green coloration. *Hoplia argentea* is widespread in Europe and Asia [18]. It inhabits open mountain meadows, where it is usually found on herbaceous plants, from May to September. The larvae live in the soil, where they feed on the roots of various plants during the summer, while they hibernate during the winter and come out in the spring as adults [36]. The green coloration usually has a cryptic role in insects providing very effective camouflage during the feeding on green vegetation [37]. This is also supported by correlation of reflection spectra of elytra and green leaves [17, 37]. In the biology of *H. argentea*, the mechanism of humidity-induced color change could also be important for camouflage when insect descends from vegetation to moist soil, especially for females when lay eggs. The elytra are soaked with moisture and change color to brown–red, which blend the insect with the soil environment and thus protects from predators. On the other hand, in terms of heat conductivity, water is classified as a poor thermal conductor and acts as an insulator by resisting the flow of heat through it. Thus, elytra soaking may play a role in preventing heat loss and insect thermoregulation. This same trait has also been found in some other species of beetles, such as *Dynastes hercules* [7].

From the more practical point of view, we have found that scales of *H. argentea* can be quickly and completely filled with many liquids (e.g., water, alcohol, glycerin). Dissolved active compounds can be thus introduced into the scales, which can be further used as vehicles for controlled release of pharmaceuticals in medicine or fluorescent sensors if filled with appropriate dyes. In particular, dynamic percolation patterns have drawn our attention, as we found them unique for each scale, and quite reproducible in repeated experiments. In this way, scales can be regarded as physically unclonable functions and used in security applications, linking optics and microfluidics in a complex interplay, virtually impossible to forge or duplicate. Also, extremely high pressures built inside the scales open up the possibility to be used for pumping of gasses and their filtration.

## Materials and methods

### Insect

Specimens of *Hoplia argentea* were collected in June 2022 on the mountain Borski Stol, Serbia (leg. S. Ćurčić & N. Vesović). We had four male and two female individuals at our disposal. Also, we had one *H. coerulea* individual which we got from colleagues from Belgium. Samples were stored in dry and dark conditions before measurements, which were performed in lab conditions. The species are not endangered according to the IUCN categorization.

### Morphological and optical analysis

A stereomicroscope (STEBA600, Colo Lab Experts, Slovenia) with maximum magnification up to 180X, eyepiece 20X, auxiliary objective 2X, working distance 100 mm, reflection and transmission mode, and equipped with a digital camera (Canon EOS 50D, Tokyo, Japan) was used to examine the anatomy of the whole insect. The optical characteristics of the elytra and setae were analysed on a trinocular microscope (MET104, Colo Lab Experts, Slovenia) (maximum magnification 400X, polarization set, objectives Plan Achromatic POL Polarizing 10X/20X/40X).

The corresponding wing spectra were recorded in reflection using a fiber optic spectrometer (manufactured by Ocean Optics, HR2000CG-UV-NIR), with a 400-μm core diameter fiber. A halogen lamp was used as a light source, and spectra were referenced to a standard white surface.

A field emission gun scanning electron microscope (FEGSEM) (MiraSystem, TESCAN, Czech Republic) was used for ultrastructural analysis. Prior to analysis, insect elytra were removed and placed on an aluminium mount and coated with a thin layer (5–10 nm) of gold palladium (AuPd), using a SC7620 Mini Sputter Coater (Quorum Technologies Ltd., UK).

Micro-computed tomography (micro-CT) was employed to view the overall anatomy of the beetle and measure the thickness of the elytra and position of the scales. We had at our disposal the Skyscan 1172 system, manufactured by Bruker. To ensure the optimum signal/noise ratio during micro-CT imaging, the specimens were scanned without filter, with scanning parameters set as follows: 40 kV, 244µA, 530 ms, rotation step 0.2° (pixel size 13.5 µm).

Nonlinear Laser Scanning Microscope (NLSM) was constructed in-house [38] and was used to analyze three-dimensional (3D) structure of elytra, using two-photon excited fluorescence (TPEF) of chitin. We used femto-second laser (Ti–sapphire laser (CoherentMira 900-F), pumped by a 10-W (Coherent Verdi V10) laser at 532 nm, generates 160-fs pulses at a 76-MHz repetition rate within the 700- to 1000-nm tuning rang) to generate two-photon excitation fluorescence (TPEF). We used Carl Zeiss Planachromat, 20 × 0.8 NA microscope objective. Only natural autofluorescence of the chitin was detected and the best signal was obtained at 730 nm. VolView 3.4, open-source software (by Kitware,Inc.), was used for three-dimensional (3-D) visualization of a set of slices (either using volume rendering or maximum intensity projection algorithms).

### Optical modeling

Most laws of physics are usually expressed using partial differential equations. Solving them for most problems is not possible with analytical methods. That is why physical discretization is introduced, where space is usually divided into a finite number of elements (unit cells) of finite dimensions, so that partial differential equations can be solved by numerical methods. With the help of FEM software, it is possible to predict the propagation of electromagnetic waves in different media, as well as to calculate the reflectivity or transmissivity of the media by solving Maxwell's equations with the precise definition of initial and boundary conditions.

**Fig. 13:**
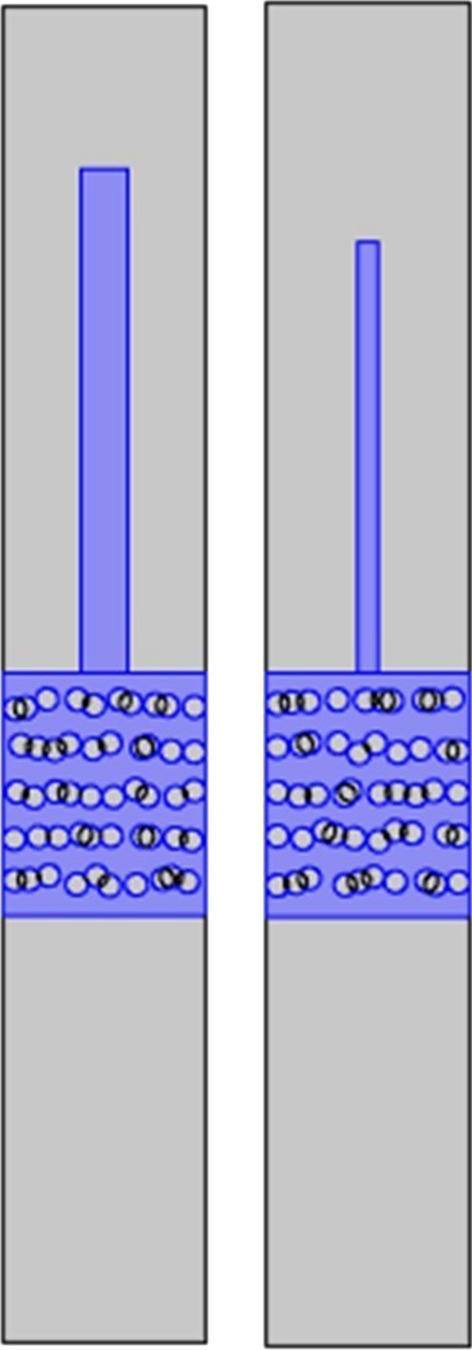
Two different models used to study optical properties of *H. argentea* scales (chitin is depicted in purple color, while air is gray). Air filled layers and fiber-like protrusions were stochastically varied to account for irregularity and variability of real, biological structures

Here we used a detailed model (Fig. 13) and finite element method to analyze the reflective properties of *H. argentea* elytron. In the model it is assumed that the structure is irradiated by a plane electromagnetic wave with different wavelengths (380–800 nm) and at different angles (0°, 20° and 40°). However, we were not able to model stochastic characteristics of scales directly as this would result in excessively complex structure difficult to calculate in a reasonable time. That is why we made 50 slightly different models, expressing to a certain extent variability of natural structures. The same calculation of spectral and angular properties was repeated for each model and the resulting spectra were averaged.

Based on the SEM images of insects, the distance between setae filaments was fixed at 1 µm, while height varied between 2–3 µm and width between 0.2 and 0.4 µm. The dimension of air—filled layers was taken to be 100–110 nm, while the distance between layers was 95 ± 30 nm. The optical characteristics of the biological structure are taken from the available literature: the refractive index is n = 1.56 [9]. The unit cell in finite element analysis was 1 µm wide, which corresponds to the distance between the setae filaments, with periodic boundary conditions. Average mesh size was manually set to 55 nm except in the case of the smallest structures where the mesh density was finer. Complete mesh consists of 45,016 domain elements and 3223 boundary elements. The calculation time per model on our computer was approximately 10 min.

### Microfluidic system

We start this section with a brief introduction to basic notions of fluidics for uninitiated readers. Interfaces between water and walls of micro and nano channels of *H. argentea* induce the water flow (capillary action). Balance between surface tension (quantified through coefficient γ) and adhesive forces (quantified through water contact angle) produce pressure difference inside the fluid, according to Young–Laplace equation. If sufficiently high, as in *H. argentea*, this pressure forces entrapped air to permeate through the submicron thin walls of the scale. Permeation is yet another complex process which includes absorption of air molecules inside the scale wall, their diffusion (Fick’s law) and desorption on the other side of the wall. Finally, diffused molecules dissolve in the water in proportion to its pressure (Henry’s law).

Additionally, nanochannels of each scale represent a random labyrinth and water flows through in a manner described by percolation theory–a branch of statistical physics describing, among others, phenomena of fluid flow through randomly connected networks. In this case, Darcy’s law connects volume flow with a fluid pressure drop.

As a result, we found the processes inside the *H. argentea* impossible to analyze fully and exactly, but approximations enabled us to broadly understand the optofluidic properties of insect’s elytra.

The amazing intricacy of optofluidic behaviour convinced us that each scale is unique structure, different from all the others. Dynamics and pattern of fluid flow and the resulting color change are easily observable, and cannot be replicated in another scale or by any artificial means. This makes them ideal candidates for physically unclonable function (PUF) devices and associated security applications.

**Fig. 14:**
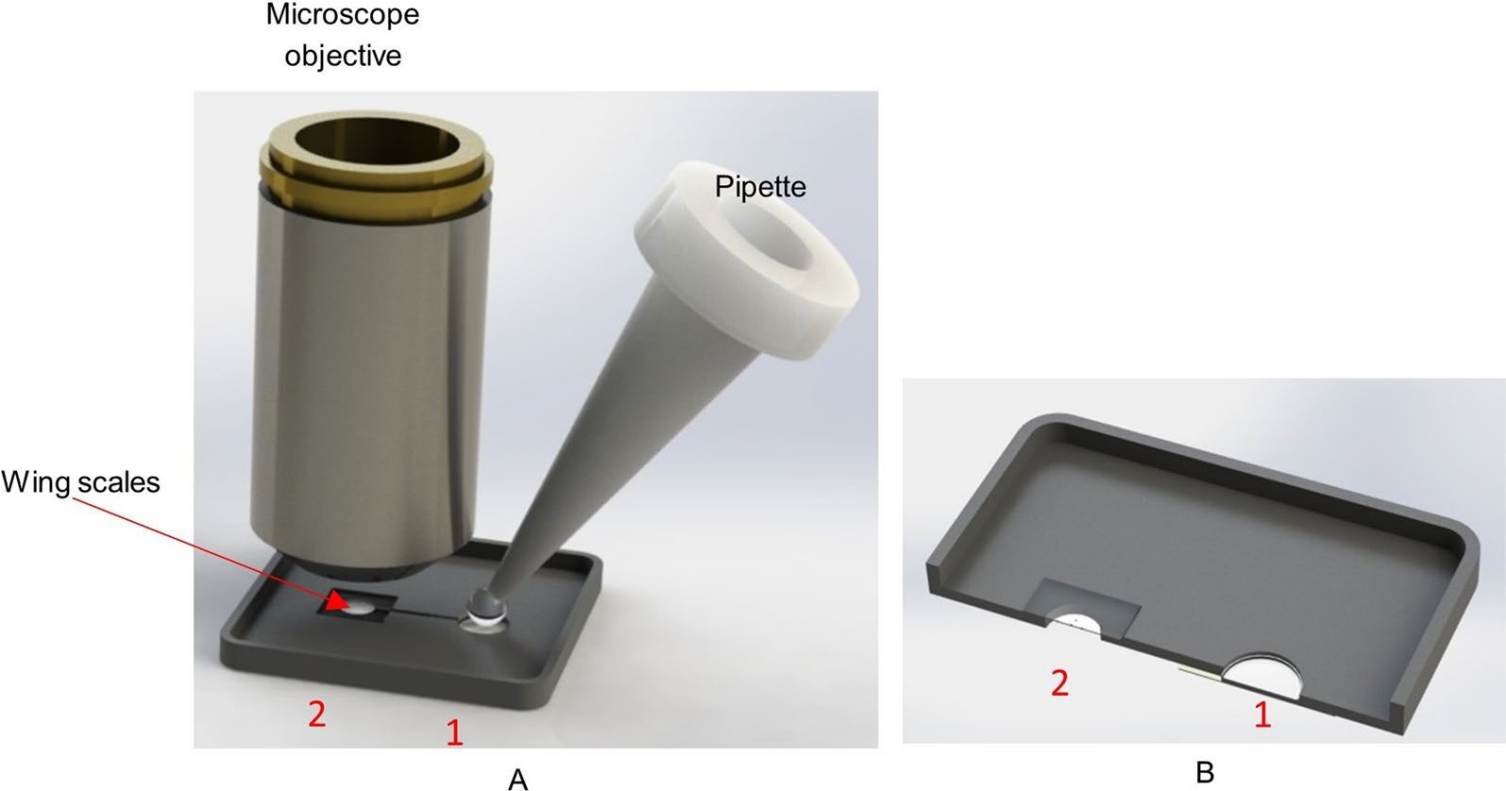
A system used for studying microfluidic properties of scales. **A**. A lab-on-a-chip system. **B**. a cross section of the lab-on-a-chip. 1 is a chamber where a water droplet is input and 2 is a chamber where *H. argentea* scales are placed

In order to study microfluidic properties of *H. argentea* scales in a controllable manner we have designed a simple, open-channel, lab-on-a-chip system (LoC—see Fig. 14). Two chambers are connected via 200 µm width channel. A bottom (floor) of the chamber 1 and the top (ceiling) of the chamber 2 are covered with a transparent pressure-sensitive (PS) tape. Initially, both chambers are empty and the wing scales are attached to the top of the chamber 2. A 20–30 micro-liter drops of water are poured in the chamber. Capillary forces drive water through the channel into the chamber 2, where scales are gradually flooded by the water. It should be noted that the scales are microscopically observed through the ceiling of the chamber 1. This enables constant position of scales with respect to the focal plane of the microscope objective (since the water is below the scales and scales are firmly attached to the PS tape). Essentially, water flow is upside down, constrained only by the surface tension and capillary forces. The experiment starts after placing the LoC system under the microscope, and adjusting the sharpness of the image.

## Supplementary Information

**Additional file 1**: Flow of water through the elytra of *H. argentea.*

**Additional file 2**: Capillary flow of water into the single *H. argentea* scale.

**Additional file 3**: Capillary flow of water out of the single *H. argentea* scale.

## Data Availability

The data that support the findings are included as electronic supplementary material.
